# Genetic analysis of agronomic traits in elite sugarcane (*Saccharum spp*.) germplasm

**DOI:** 10.1371/journal.pone.0233752

**Published:** 2020-06-11

**Authors:** Fenggang Zan, Yuebin Zhang, Zhuandi Wu, Jun Zhao, Caiwen Wu, Yong Zhao, Xuekuan Chen, Liping Zhao, Wei Qin, Li Yao, Hongming Xia, Peifang Zhao, Kun Yang, Jiayong Liu, Xiping Yang

**Affiliations:** 1 Yunnan Province Key Laboratory of Sugarcane Genetic Improvement/ Sugarcane Research Institute, Yunnan Academy of Agricultural Sciences, Kaiyuan, Yunnan Province, China; 2 Guangxi Key Lab of Sugarcane Biology, Guangxi University, Nanning, Guangxi, China; 3 Agronomy Department, University of Florida, Gainesville, FL, United States of America; Louisiana State University, UNITED STATES

## Abstract

Sugarcane (*Saccharum* spp.) is an important economic crop, supplying up to 80% of the table sugar and ~60% of bio-ethanol worldwide. Due to population growth and dwindling fossil-fuel reserves, the demand for sugar and bio-ethanol requires significant improvement in sugarcane production. Breeding sugarcane cultivars with high-performance agronomic traits is undoubtedly the most efficient way to achieve this goal. Therefore, evaluating agronomic traits and dissecting underlying loci are critically important for this aim steps in providing genetic resources and molecular markers for selection. In this study, we assembled a diversity panel of 236 elite sugarcane germplasms originally collected from 12 countries. We evaluated 28 agronomic traits in the diversity panel with three replicates. The diversity panel was genotyped using amplified fragment length polymorphism markers, and a total of 1,359 markers were generated. Through the genome-wide association study, we identified three markers significantly associated with three traits evaluated at a stringent threshold (*P* < 0.05 after Bonferroni correction). The genotypes of the three associated markers grouped respective trait values into two distinct groups, supporting the reliability of these markers for breeding selection. Our study provides putative molecular markers linked to agronomic traits for breeding robust sugarcane cultivars. Additionally, this study emphasized the importance of sugarcane germplasm introduced from other countries and suggested that the use of these germplasms in breeding programs depends on local industrial needs.

## Introduction

As an efficient C_4_ crop, sugarcane (*Saccharum* spp.) provides up to 80% of the table sugar and ~60% of bio-ethanol worldwide [1]. The crop is mainly cultivated in tropical and sub-tropical areas in over 100 countries covering ~27.1 million ha with a total harvest of 1.9 billion metric tons/year [2–4]. Due to human population growth and dwindling fossil-fuel reserves, the demand for sugar and bio-ethanol requires significant improvement in sugarcane production. There is no doubt that breeding sugarcane cultivars with high-performance agronomics traits is an efficient way to achieve this goal. Therefore, assembling a large germplasm panel, surveying important agronomic traits, and dissecting underlying loci controlling these traits are critically important steps in the development of genomic resources and tools for sugarcane breeding programs working to reach this goal.

Modern sugarcane hybrids are derived from inter-specific hybridization between *S*. *spontaneum* (2n = 40–128, x = 8) and *S*. *officinarum* (2n = 80, x = 10) followed by several backcrosses with *S*. *officinarum* [5, 6]. Sugarcane breeding programs have suffered from a narrow genetic base in sugarcane hybrids because only a handful of ancestral clones were involved in the initial hybridizations. Furthermore, this issue has been exacerbated because only a few popular sugarcane hybrids have been widely used as parental lines [7–9]. For example, more than 90% of sugarcane cultivars in the USA can be traced back to 10 ancestral clones [9, 10]. In China, a single cultivar ROC22 occupied 50–60% of the planting areas and was the most frequently used parental material in breeding programs [11, 12]. Therefore, a broadened genetic base for sugarcane cultivars could improve sugarcane production, and reduce potentially devastating disease outbreaks because of the planting of cultivars with similar backgrounds in large areas. Importing and using elite sugarcane clones from other countries could significantly hasten the achievement of this goal for two major reasons. First, elite sugarcane clones derived from different breeding programs likely have different genetic backgrounds. Second, these elite sugarcane clones were selected for high production, and have enriched robust agronomic traits.

Modern sugarcane hybrids have a chromosome number of 100–130 (2n = 10–12x) with an estimated genome size of approximately 10 Gbp [13]. Because of sugarcane’s huge genome size and high polyploidy level (primarily aneuploidy), a huge number of markers are required to evenly cover the whole genome. Amplified fragment length polymorphism (AFLP) is a PCR-based genotyping technique, which can generate large numbers of markers using a small amount of DNA. In addition, AFLP does not rely on expensive equipment and can be easily applied in almost any lab. In sugarcane study, AFLP has been successfully used to assess genetic diversity, and conduct linkage mapping, quantitative trait loci mapping and genome-wide association studies (GWAS) [14–19]. In addition, AFLP markers can be converted into high-throughput markers [20], which could expedite the application of AFLP markers linked to important agronomic traits in sugarcane breeding programs.

In this study, we assembled a sugarcane diversity panel consisting of 236 elite sugarcane germplasms originating from 12 countries. There were two objectives for the current study: 1) to evaluate the performance of these elite sugarcane germplasms and select breeding material with robust agronomic traits for breeding programs in China; and 2) to identify markers linked to agronomic traits. Consequently, we evaluated 28 agronomic traits in this diversity panel in China in replicated trials, and genotyped the whole population using AFLP. At a stringent threshold, three markers were identified that were significantly associated with three traits.

## Materials and methods

### Plants in the association panel

A sugarcane diversity panel was assembled, which consisted of 236 accessions (S1 Table), including 39 from Australia, 13 from Brazil, 81 from France, 30 from the Philippines, two from Cuba, one from Malaysia, 16 from the USA, three from Bangladesh, 14 from Mexico, five from Republic of the Sudan, two from India, and 30 from China. All these sugarcane accessions were chosen from the collections of China’s National Germplasm Repository of Sugarcane, provided by the National Infrastructure for Crop Germplasm Resources.

### Agronomic traits evaluation

The sugarcane diversity panel with 236 accessions was established using a randomized complete block design with three replicates in a field nursery at Sugarcane Research Institute, Yunnan Academy of Agricultural Sciences at Kaiyuan, Yunnan in March 2010. Plot length was 3 m with 1 m between-row spacing. From seedling to maturity of the seed cane, we surveyed seven continuous traits, including fiber content (Fiber), sugar content (Sugar), germination rate (GerminationR), tiller rate (TillerR), number of productive tillers (ProTiller), plant height (Height) and stalk diameter (Diameter). Additionally, we surveyed 21 categorical traits, including aerial root (AeriRoot), stalk shape (StaShape), internode shape (InterShape), internode color unexposed (InterColUn), internode color exposed (InterColEx), wax band (WaxBand), corky patches (CorkyPat), growth crack (GroCrack), growth ring shape (GroRingShape), root primordia (RootPri), bud shape (BudShape), bud placement (BudPlace), bud furrow (BudFurrow), angle of lamina to culm (AngLamCul), lamina color (LamCol), sheath detached from culm (Sheath), hair group 57 (Hair), shape of inner auricle (ShapeInAur), shape of outer auricle (ShapeOuAur), pith, and cotton. A short description is included to explain these traits in Table 1. Traits were evaluated following general procedures [21]. Specifically, fiber content and sugar content were measured in January 2011. Germination rate was defined as the percentage of germinated buds of all planted buds, and the tiller rate was calculated based on the observation of tillers after completion of tillering. The productive tillers were those greater than 1 m stalks. For all traits, all measurements or observations were performed on five stalks for each replicate. An analysis of variance (ANOVA) was performed using the *agricolae* package in R [22, 23]. Average values of the three replicates were used for pairwise Pearson’s correlation calculations using the *Hmisc* package in R [24] and subsequent GWAS.

**Table 1:** The 28 agronomic traits evaluated in the sugarcane diversity panel.

Trait	Abbreviation	Continuous or categorical value	Number of levels	Description
Aerial root	AeriRoot	Categorical	2	1 (no), 2 (yes)
Stalk shape	StaShape	Categorical	2	1 (upright), 2 (bending)
Internode shape	InterShape	Categorical	6	1 (cylindrical), 2 (waist drum), 3(thin waist), 4 (conical), 5 (Inverted conical), 6 (curved shape)
Internode color unexposed to light	InterColUn	Categorical	8	1 (pink), 2 (purple-red), 3 (dark purple), 4 (yellow), 5 (yellow-green), 6 (dark green), 7 (purple green stripes), 8 (purple yellow stripes)
Internode color exposed to light	InterColEx	Categorical	8	1 (pink), 2 (purple-red), 3 (dark purple), 4 (yellow), 5 (yellow-green), 6 (dark green), 7 (purple green stripes), 8 (purple yellow stripes)
Wax band	WaxBand	Categorical	3	1 (no), 2 (thin) 3 (thick)
Corky patch	CorkyPat	Categorical	3	1 (no), 2 (stripe) 3 (Plaque)
Growth crack	GroCrack	Categorical	3	1 (no), 2 (shallow) 3 (deep)
Growth ring shape	GroRingShape	Categorical	2	1 (protruding), 2 (not protruding)
Root primordia	RootPri	Categorical	2	1 (line-up), 2 (irregular)
Bud shape	BudShape	Categorical	9	1 (triangle), 2 (oval), 3 (obovate), 4 (pentagon), 5 (diamond), 6 (round), 7 (square), 8 (rectangle), 9 (beak shape)
Bud placement	BudPlace	Categorical	3	1 (up), 2 (middle) 3 (down)
Bud furrow	BudFurrow	Categorical	3	1 (no), 2 (shallow), 3 (deep)
Angle of lamina to culm	AngLamCul	Categorical	3	1 (scatter), 2 (drooping), 3 (straight)
Lamina color	LamCol	Categorical	4	1 (green), 2 (yellow-green), 3 (dark green), 4 (red purple)
Sheath detached from culm	Sheath	Categorical	3	1 (shedding), 2 (loose), 3 (tight)
Hair group 57	Hair	Categorical	4	1 (no), 2 (few), 3 (intermediate), 4 (many)
Shape of inner auricle	ShapeInAur	Categorical	6	1 (degenerated), 2 (triangle), 3 (barb), 4 (sickle shape), 5 (needle), 6 (hook)
Shape of outer auricle	ShapeOuAur	Categorical	6	1 (degenerated), 2 (triangle), 3 (barb), 4 (sickle shape), 5 (needle), 6 (hook)
pith	Pith	Categorical	4	1 (no), 2 (soft), 3 (intermediate), 4 (heavy)
Cotton	Cotton	Categorical	2	1 (no), 2 (yes)
Fiber content	Fiber	Continuous	Continuous	Fiber content (%)
Sugar content	Sugar	Continuous	Continuous	Sucrose in cane (%)
Germination rate	GerminationR	Continuous	Continuous	Germination percentage
Tiler rate	TillerR	Continuous	Continuous	Tiller rate (%)
Plant height	Height	Continuous	Continuous	Plant height (cm)
Stalk diameter	Diameter	Continuous	Continuous	Stalk diameter (cm)
Number of productive tillers	ProTiller	Continuous	Continuous	number of productive tillers

### AFLP genotyping

Young leaves were collected from five stalks of each accession and combined for DNA extraction by using the cetyltrimethyl ammonium bromide (CTAB) method [25]. The quality and concentration of extracted DNA were evaluated by 1% agarose gel electrophoresis. DNA with good quality was diluted to 20 ng μL^-1^, and stored at −20°C.

AFLP procedures were adapted from the AFLP protocol previously described [26] with minor adjustments. In brief, genomic DNA was digested using *Eco*RI and *Mse*I at 37°C for 14 h. Ligation of the completely digested genomic DNA and adapters of *Eco*RI and *Mse*I was performed at 37°C for 2 h. The ligation product was diluted 10 times as a pre-selective PCR template, which was conducted using *Eco*RI and *Mse*I pre-selective primers (with a selective nucleotide) in an Eppendorf Master Cycle Gradient thermal cycler (Eppendorf, Hamburg, Germany) with 20 cycles at 94°C for 30 s, 56°C for 60 s, and 70°C for 60 s. Selective PCR was conducted using pre-selective PCR products (diluted 10 times) as a template using *Eco*RI and *Mse*I selective primers (with three selective nucleotides) first with 13 cycles at 94°C for 30 s, 65°C (reduced by 0.7°C for the next 12 cycles), and 70°C for 60 s, and then with 23 cycles at 94°C for 30 s, 56°C for 60 s and 70°C for 60 s. The selective PCR products were denatured at 95°C for 10 min and then electrophoresed on a 5% denaturing polyacrylamide gel. The AFLP amplified bands were scored as 1 (presence) or 0 (absence) (S1 Fig).

### GWAS

The genetic structure of the diversity panel was assessed using the default settings in the discriminant analysis of principal components (DAPC) implemented in the *adegenet* package in R [27, 28]. We further used phylogenetic analysis to infer genetic relationships among the diversity panel accessions using Molecular Evolutionary Genetics Analysis version 6.0 (MEGA 6.0) [29] and the neighbor-joining method. The pairwise genetic distances for the 236 accessions were obtained by MEGA 6.0 using the p-distance method. Average phenotypic values of the three replicates were used in the GWAS, which was conducted in GWASpoly [30] using the Q + K mixed model. GWASpoly was designed to perform the GWAS while taking allelic dosages and interactions into consideration. The software was initially tested in a tetraploid population, but can be extended to other polyploids and has been successfully applied in numerous species, including sugarcane [31].

## Results

### Agronomic traits

**Fig 1:**
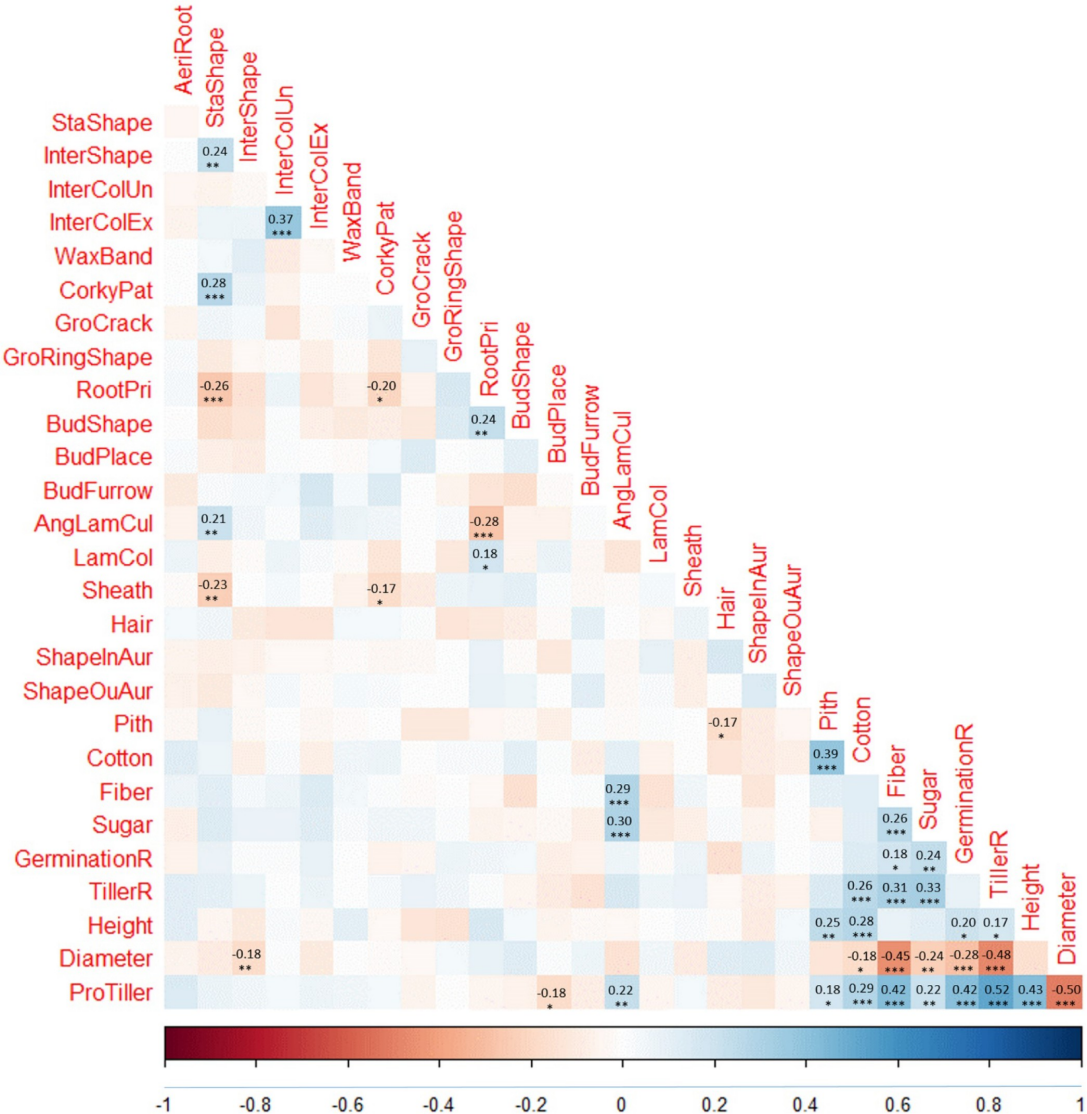
Pairwise pearson's correlation coefficients for the 28 agronomic traits in the sugarcane diversity panel. *P < 0.01; **P < 0.001; ***P < 0.0001.

In total, 28 agronomic traits were evaluated in the sugarcane diversity panel with three replicates, including 21 categorical and seven continuous traits (Table 1). According to the pairwise Pearson’s correlation coefficients for the 28 traits (392 pairs), 41 pairs of traits (out of 378 pairs) were highly correlated (*P* < 0.01) (Fig 1). Among the highly correlated traits, 13 pairs were between categorical traits (of 210 pairs), 17 pairs were between continuous traits (of 21 pairs), and 11 pairs were between categorical and continuous traits (of 147 pairs). The continuous traits tended to be highly correlated compared with that of categorical traits, and categorical with continuous traits. Continuous traits controlled by multiple genes likely shared genes or regulation pathways and this might explain the phenomenon. Specifically, between categorical traits, StaShape was correlated with five traits (InterShape, CorkyPat, RootPri, AngLamCul and Sheath), RootPri with three traits (BudShape, AngLamCul and LamCol), CorkyPat with two traits (RootPri and Sheath), InterShape with InterColEx, and Pith with Cotton. Between categorical and continuous traits, AngLamCul was positively correlated with Fiber, Sugar, and ProTiller, Cotton positively correlated with TillerR, Height, and ProTiller, and Pith was positively correlated with Height and ProTiller, whereas both InterShape and Cotton were negatively correlated with Diameter, and BudPlace was negatively correlated with ProTiller. Between continuous traits, all were highly correlated except for four pairs of traits, including Height with Fiber, Sugar, and Diameter, and GerminationR with TillerR. Among the highly correlated continuous traits, five pairs were negatively correlated, including Diameter with Fiber, Sugar, GerminationR, TillerR, and ProTiller, whereas all others were positively correlated.

**Fig 2:**
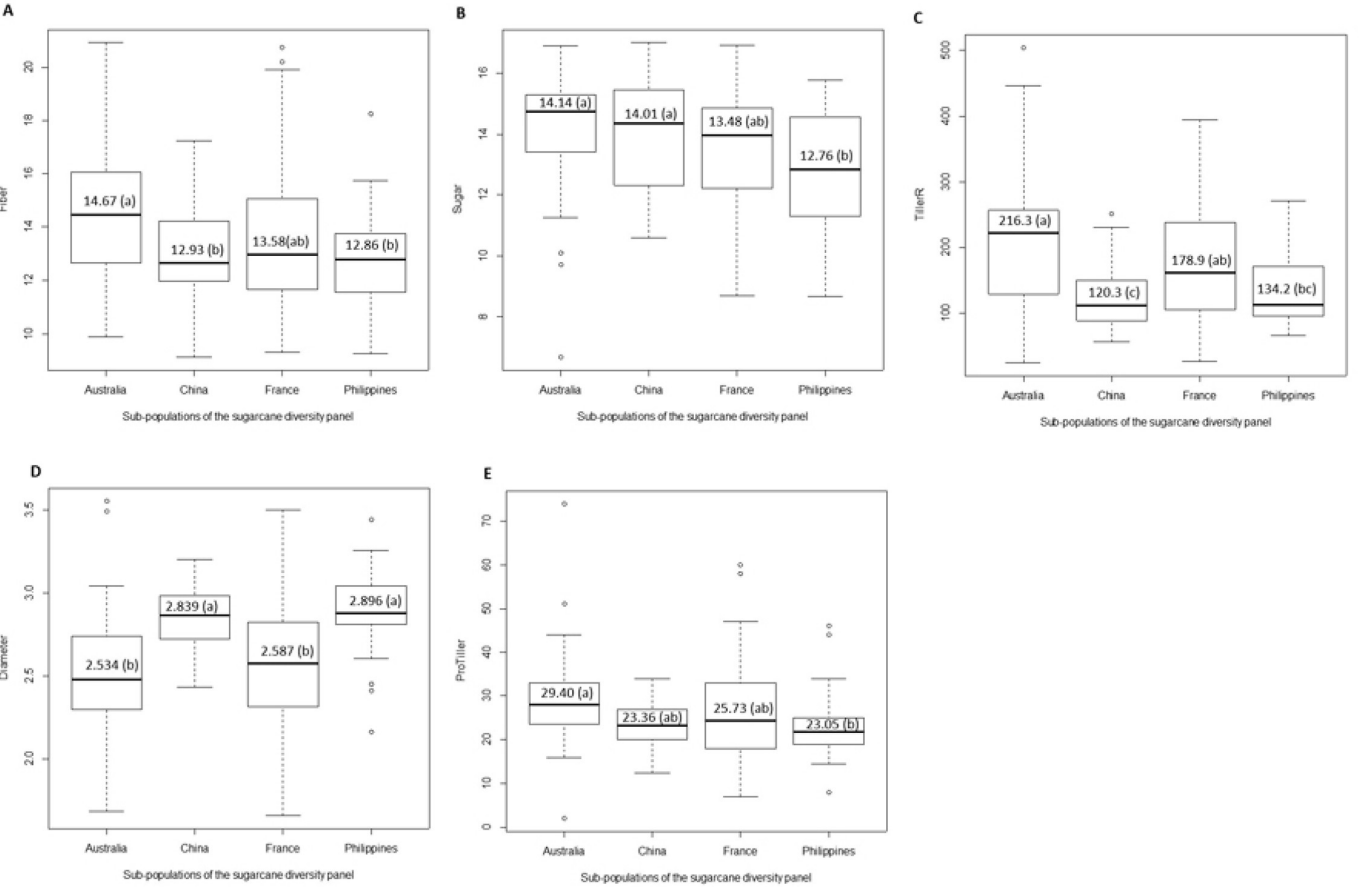
Summary of continuous traits in the diversity panel that were significantly different among the four counties with at least 30 accessions. A) Fiber; B) Sugar; C) TillerR; D) Diameter; and E) ProTiller.

We compared the seven continuous traits among countries with at least 30 accessions collected in the diversity panel, i.e., Australia (39 accessions), China (30 accessions), France (81 accessions), and the Philippines (30 accessions). Five traits including Fiber, Sugar, TillerR, Diameter, and ProTiller, exhibited a significant difference among the four countries (*P* < 0.05) (Fig 2). No difference was observed for GerminationR and Height. The accessions from Australia had much better performance for Fiber, Sugar, TillerR, and ProTiller. However, Diameter in accessions from Australia was lower compared with that from other countries. The accessions from China had a high value for Diameter, medium for ProTiller, but low for Fiber and TillerR. The performance of accessions from France for the seven continuous traits was similar to that of Australia, whereas the performance of accessions from the Philippines was similar to that of China but with lower Sugar. The results likely reflect differences in selection criteria were applied in different breeding programs or these elite cultivars have different genotype by environment interactions while growing in China.

### AFLP genotyping and population structure

**Fig 3:**
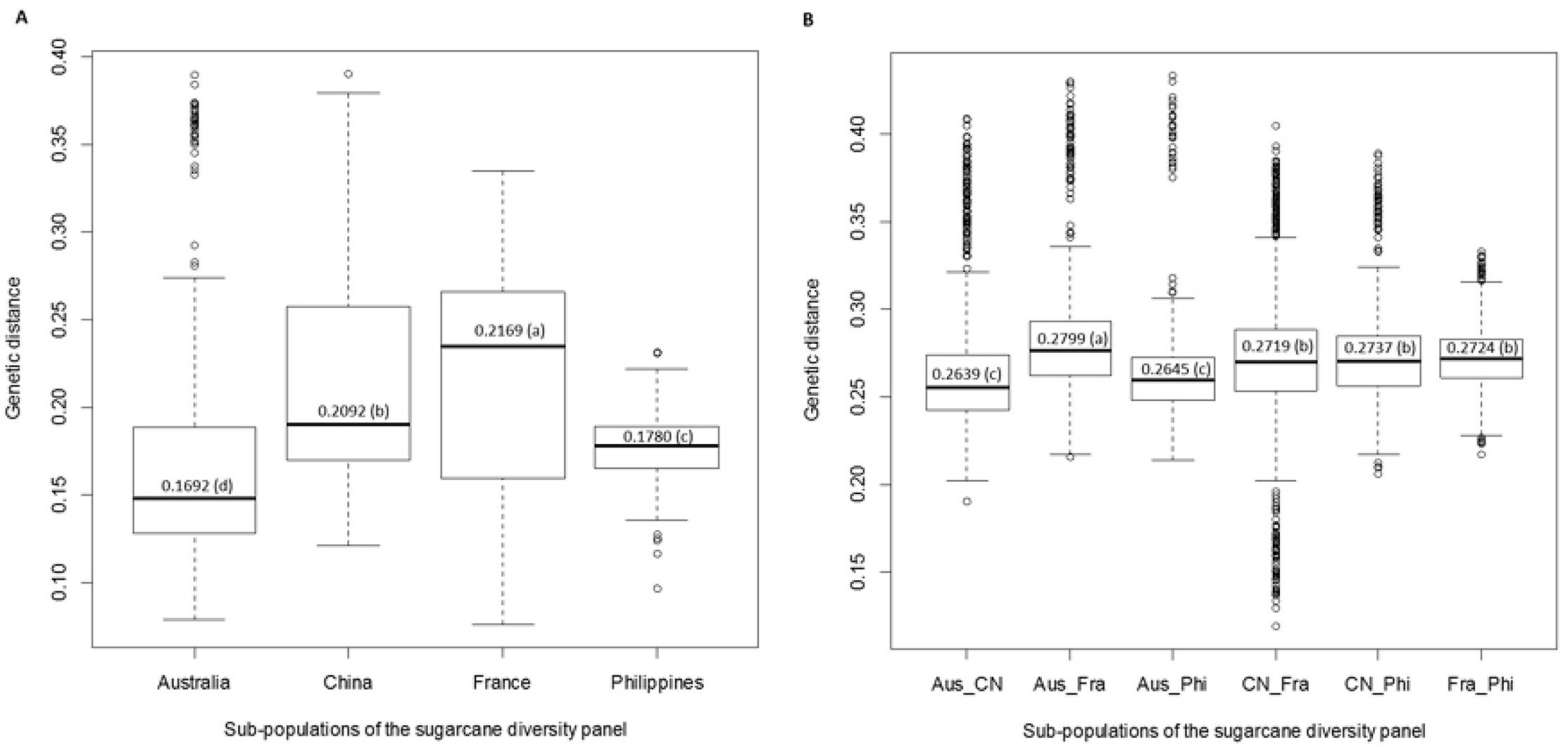
Genetic distance in sugarcane diversity. A) Genetic distance among the four countries with at least 30 accessions; B) Pairwise genetic distance among the four countries. Aus = Australia; CN = China; Fra = France and Phi = the Philippines.

Among the 10 primer combinations evaluated, a total of 1,359 polymorphic fragments were identified in the diversity panel (S2 Table). The average number of fragments was 136, ranging from 103 (E-ACT/M-CAG) to 178 (E-ACT/ M-CAA). We calculated the genetic distance among the four countries with at least 30 accessions. The genetic distance was significantly different among the four countries (*P* < 0.0001) with an average genetic distance of 0.1692, 0.2092, 0.2169, and 0.1780 for accessions from Australia, China, France, and the Philippines, respectively (Fig 3). The accessions from France had the greatest genetic distance followed by that of China and the Philippines, whereas the accessions from Australia had the smallest distance. The pairwise genetic distance was also significantly different among the four countries (*P* < 0.0001) with the greatest distance between the accessions from Australia and France, and the smallest distance between Australia and China (Fig 3).

**Fig 4:**
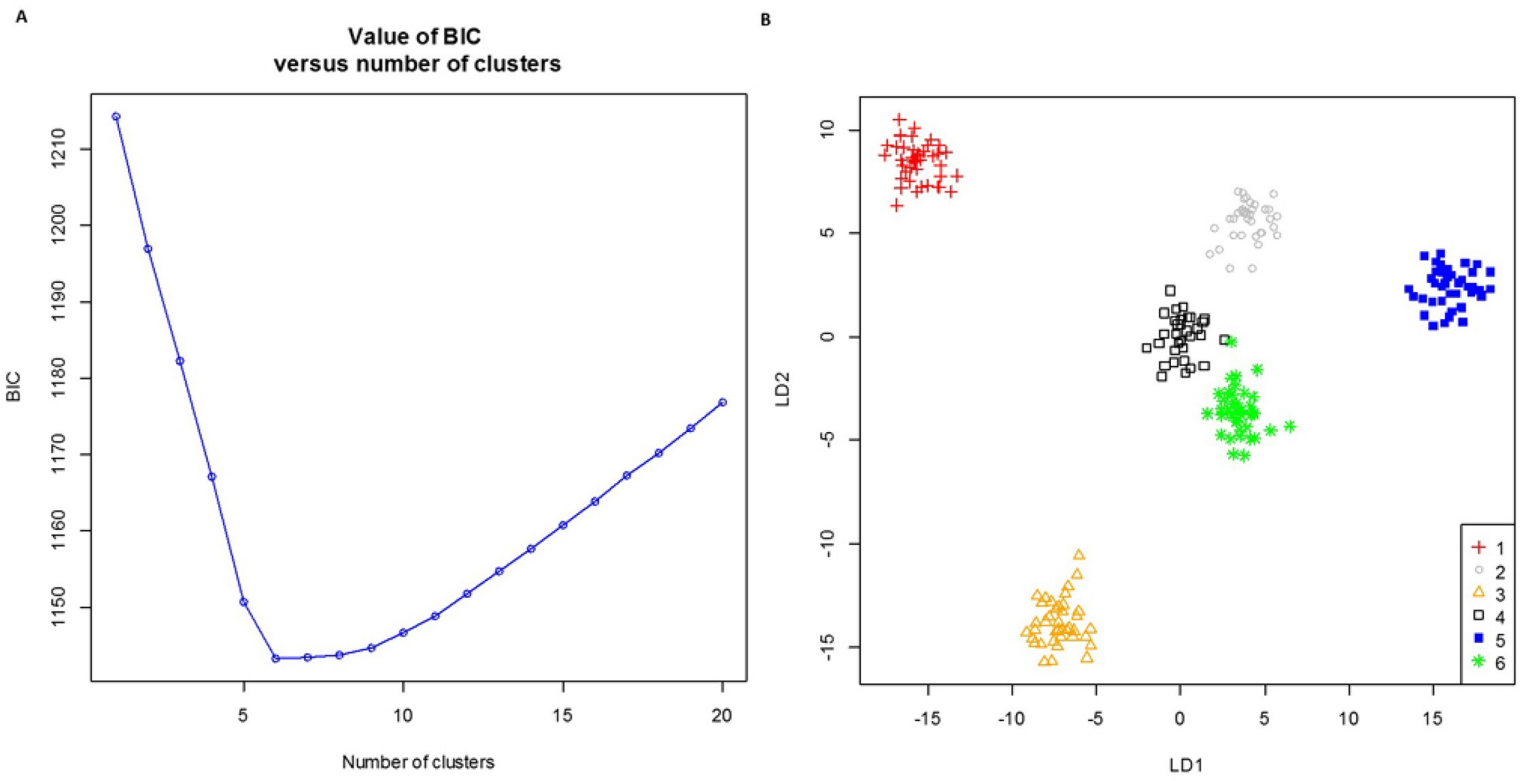
Population structures of the sugarcane diversity panel. A) Bayesian Information Criteria (BIC) vs. the number of clusters in k-means clustering. B) Projection of the sugarcane diversity panel using the first two linear discriminants (LDs).

We assessed the population structure of the sugarcane diversity panel with DAPC. We selected *k* = 6 to represent the number of population groups in the diversity panel, which minimized the Bayesian Information Criteria (BIC) value (Fig 4A). The diversity panel was clearly split into six groups with the number of accessions from 32 (group 4) to 44 (group 1) (Fig 4B and S1 Table). Accessions from France were assigned to group 1 (42 accessions from France and two from China) and group 3 (all 39 accessions from France). Group 2 included 34 accessions from Australia and four from the USA. Group 5 included 30 accessions from the Philippines and 12 from the USA. Group 6 included 13 accessions from Brazil and 28 from China. Group 4 included 32 accessions originally from seven countries, i.e., five accession from Australia, three from Bangladesh, two from Cuba, two from India, one from Malaysia, 14 from Mexico and five from the Republic of the Sudan. The observation of accessions from different countries assigned in the same group showed that the same or similar germplasms were probably used in as parental materials in different breeding programs. We further performed phylogenetic analysis on the same diversity panel, and the clusters of accessions were mostly consistent with the results from DAPC (S2 Fig). Therefore, the group assignment of the diversity panel was included for the subsequent GWAS.

### GWAS

**Fig 5:**
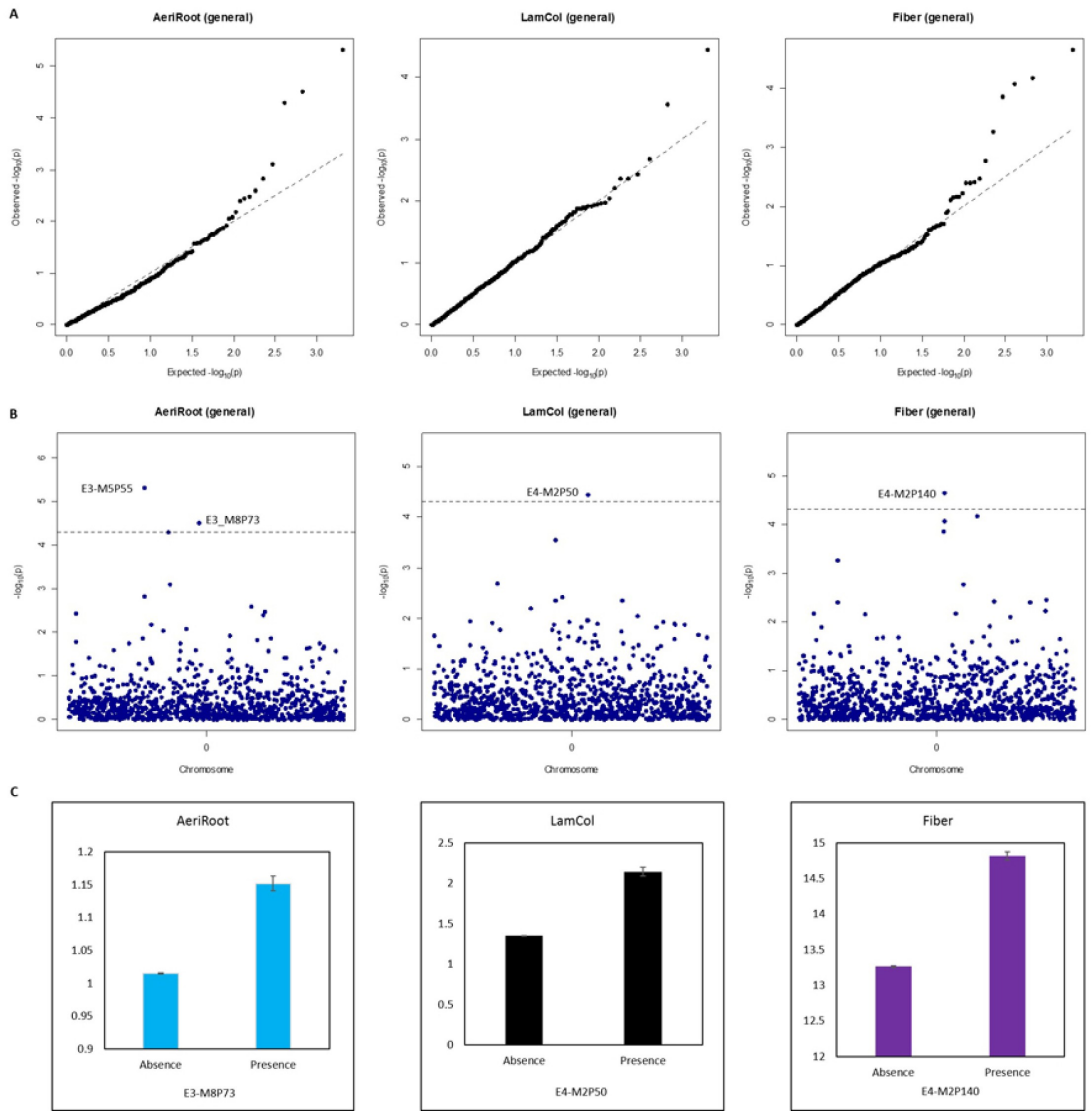
Summary of genome-wide association study in the diversity panel. A) QQ-plot; B) Manhattan plot and C) Performance of the three associated markers for grouping respective trait values. The evaluated traits of the 236 accessions were divided into two groups based genotypes of respective markers (absence vs. presence).

Applying a stringent threshold (*P* < 0.05 after Bonferroni correction), we identified four markers significantly associated with three of the traits evaluated. The QQ-plot demonstrated that DAPC could successfully reduce statistical inflation caused by the effects of population structure (Fig 5A). Among the associated markers, two were identified for AeriRoot (E3-M5P55 and E3-M8P73 with–log _10_ (*P*) of 5.32 and 4.51, respectively), one for LamCol (E4-M2P50 with -log _10_ (*P*) of 4.44), and one for Fiber (E4-M2P140 with -log _10_ (*P*) of 5.32) (Fig 5B). We checked the four associated markers for grouping of the respective trait values. Three markers (E3-M8P73, E4-M2P50 and E4-M2P140) exhibited significantly different trait values between the two genotypes of the markers (absence vs. presence) (*P* < 0.0001) (Fig 5C), indicating that they could be used as molecular markers for selection. No difference was observed for the marker E3-M5P55 on grouping AeriRoot. The presence of this marker was predominant in groups 4, 5, and 6 of the diversity panel (out of 111 accessions scored as presence, 95.5% of them were in the three groups). The association of this marker with AeriRoot was probably caused by the effects of sub-populations, and this marker was removed from the candidate markers. Therefore, in this study, we identified three markers associated with three traits. However, if a less stringent cutoff (*P* < 0.05 adjusted for the false discovery rate (FDR)) was used, 202 markers would have been identified as significantly associated with 11 traits. These results are listed in a supplemental file (S3 Table) and are not discussed here.

## Discussion

Sugarcane is the most important crop that supplies table sugar and bio-ethanol worldwide. Breeding cultivars with improved agronomic traits to increase sugarcane production is highly desirable. In this study, we assembled a diversity panel consisting of 236 elite sugarcane cultivars from 12 major sugarcane-growing countries. By assessing population structure in the diversity panel, we found that accessions from different countries could be assigned to one group. The results indicated that the same or similar parental lines from different countries were already used in sugarcane breeding programs, reflecting a certain level of germplasm exchange across the world leading to close pedigrees between accessions from different countries. Significantly different performance was observed for fiber content, sugar content, tiller rate, stalk diameter, and the number of productive tillers for the four countries mentioned above. The difference also demonstrated different selection criteria applied in sugarcane breeding programs. For example, accessions from China had large stalk diameter, high sugar content, but a relatively low tiller rate compared with accessions from Australia. This was likely indicative of the impact of sugarcane harvest methods on breeding programs. In China, sugarcane harvest mainly relies on manual harvest at current stage, and cultivars with large stalks are favored for selection. The comparison of phenotypic values and genetic diversity also suggested how the accessions introduced should be used in sugarcane breeding programs. For example, to breed sugarcane cultivars with high sugar content and tiller rate, accessions from Australia would be a good choice for sugarcane breeders in China. However, accessions from France and the Philippines had significantly greater genetic diversity compared with accessions from Australia (*P* < 0.0001), which might be helpful to broaden the genetic base of sugarcane cultivars in China. The current results emphasized the importance of sugarcane germplasm introduced from other countries, and the utilization of these germplasms should depend on the breeding programs.

With the development of sequencing technologies, array technologies and other genotyping techniques, AFLP might not be as informative as others. However, for a complex genome such as sugarcane, AFLP could efficiently generate a large number of markers. For example, applying 10 combinations of two restriction enzymes could generate 1,359 markers in this study. Additionally, it does not rely on expensive equipment and is easy to run, which makes AFLP favored by some labs, such as sugarcane breeding facilities that need to quickly and efficiently evaluate the genetic diversity of sugarcane germplasms for parental selection. Moreover, in theory, AFLP can be adapted for next generation sequencing for high-throughput genotyping, which is similar to genotyping by sequencing [32] and amplicon sequencing [33]. The candidate markers detected by AFLP can be developed as PCR-based diagnostic high-throughput markers [20].

Through GWAS, we identified three AFLP markers significantly associated with AeriRoot, LamCol and Fiber. A significant difference in the evaluated trait values was observed for two genotypes of the three associated markers, supporting that these markers could be used as molecular markers for selecting the respective traits. Fiber content is an important trait to be considered for sugarcane to serve as bioenergy feedstock, whereas AeriRoot and LamCol are characteristics to differentiate varieties. Upon validation, the associated molecular markers are powerful to identify parental lines or progeny before planting them in field, which will empower the sugarcane breeding programs by shorten breeding cycles, and save labor, space and other agricultural resources.

We did not identify any marker associated with several traits evaluated in this study. There were several possible reasons for this. First, these traits might be controlled by multiple genes with small effects that were difficult to detect. Second, the threshold to declare markers associated was too strict. For example, with a less stringent but also acceptable cutoff (*P* < 0.05 adjusted for the FDR), we could have 202 markers identified and associated with 11 of the evaluated traits. The results were reliable and deposited in a supplemental file for the sugarcane research community to explore further. The other reason was the low number of markers used to cover the large sugarcane genome. The modern sugarcane has an estimated genome size of 10 Gbp [13] and the block of linkage disequilibrium is 3.57 Mbp [34]. We estimated that approximately 2,800 markers are needed to fully cover the genome of sugarcane hybrids. The markers used in this study could only cover roughly half of the genome, and some underlying loci could not be detected in the current study.

To summarize, we evaluated multiple agronomic traits of 236 sugarcane accessions from 12 countries. Through GWAS, we identified three markers significantly associated with three traits evaluated at a stringent threshold. This study revealed the performance of agronomic traits of elite sugarcane germplasm, and provided important information for parental selection in sugarcane breeding programs in China and other countries. Upon validation, the associated markers could be further used for quick selection of desirable sugarcane parental materials and progeny.

## Supporting information

S1 Fig An example showing bands generated using AFLP (E3_M7) in part of the diversity panel, samples 1–43. The first lane was a DNA marker. The arrow indicates the bands scored as present in sample 37 and absent in samples 38 to 43.
(TIF)

S2 Fig Phylogenetic analysis of the diversity panel of 236 accessions using 1,359 AFLP markers by MEGA 6.0 with the neighbor-joining method. (TIF)

S1 Table Details of the accessions in the sugarcane diversity panel. (XLSX)

S2 Table Summary of the 10 primer combinations used in this study. (XLSX)

S3 Table Associated DNA markers identified by the genome-wide association study in the sugarcane diversity panel (*P* < 0.05 adjusted for the false discovery rate). (XLSX)

S1 Dataset (XLSX)
