# Correction: Genetic analysis of agronomic traits in elite sugarcane (*Saccharum spp*.) germplasm

**DOI:** 10.1371/journal.pone.0253363

**Published:** 2021-06-10

**Authors:** Fenggang Zan, Yuebin Zhang, Zhuandi Wu, Jun Zhao, Caiwen Wu, Yong Zhao, Xuekuan Chen, Liping Zhao, Wei Qin, Li Yao, Hongming Xia, Peifang Zhao, Kun Yang, Jiayong Liu, Xiping Yang

In the Funding statement, the grant number from the funder Less Developed Regions of the National Natural Science Foundation of China is listed incorrectly. The correct grant number is: 31660418.
