# Supplementary figures and images for: Genetic analysis of agronomic traits in elite sugarcane (Saccharum spp.) germplasm
Source: PLoS One. 2020 Jun 11;15(6):e0233752. doi: 10.1371/journal.pone.0233752 (PMC7289623; doi:10.1371/journal.pone.0233752)

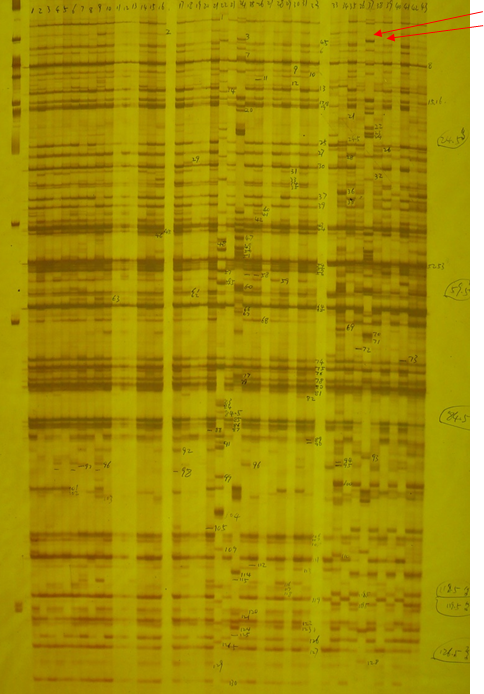

Supplement: S1 Fig — The first lane was a DNA marker. The arrow indicates the bands scored as present in sample 37 and absent in samples 38 to 43. (TIF) [file pone.0233752.s001.tif]

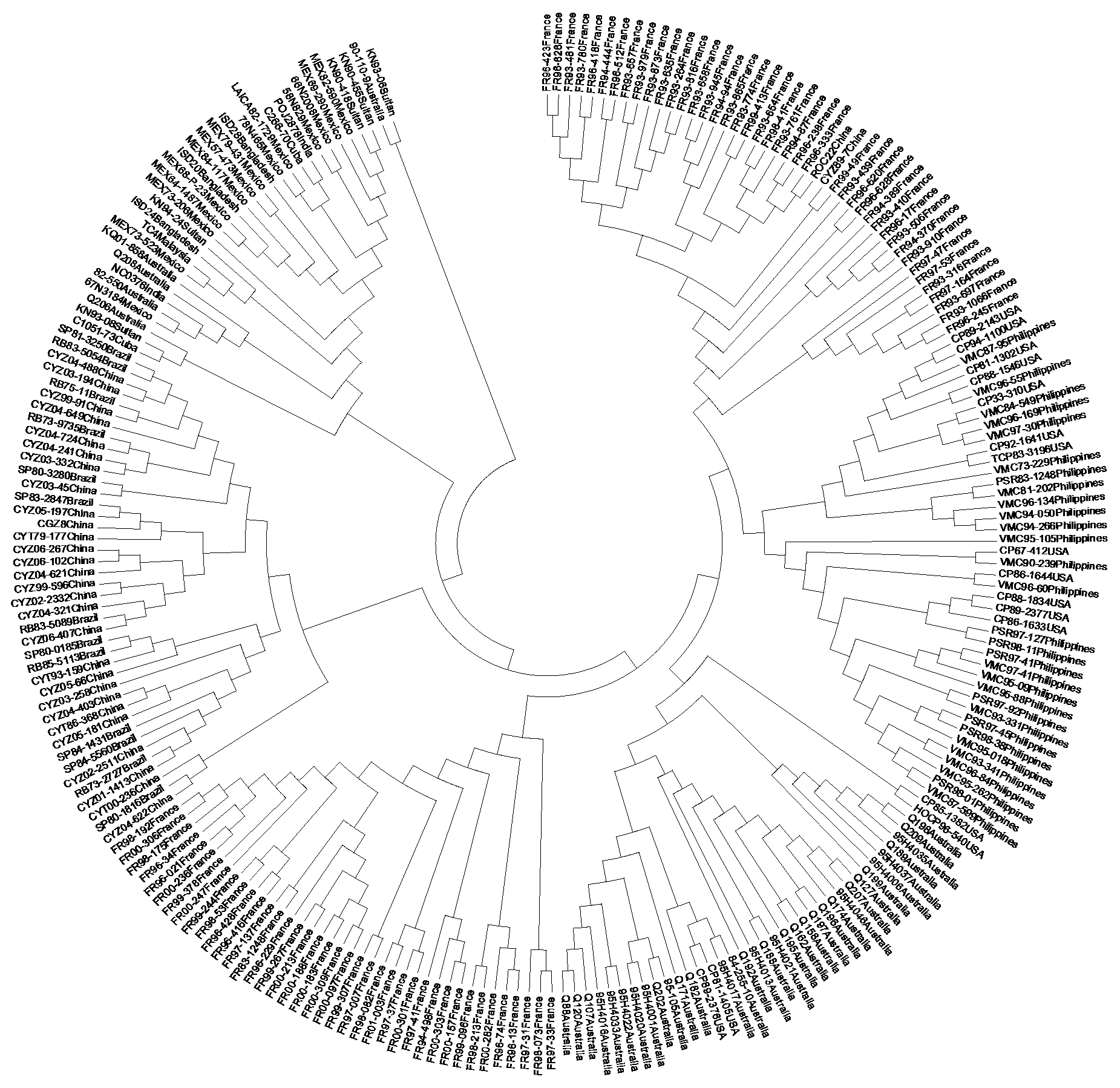

Supplement: S2 Fig — (TIF) [file pone.0233752.s002.tif]
